# Prenatal Reflective Functioning and Development of Aggression in Infancy: the Roles of Maternal Intrusiveness and Sensitivity

**DOI:** 10.1007/s10802-016-0177-1

**Published:** 2016-06-25

**Authors:** H. J. A Smaling, S. C. J. Huijbregts, K. B. van der Heijden, D. F. Hay, S. H. M. van Goozen, H. Swaab

**Affiliations:** 1 Department of Clinical Child and Adolescent Studies, Leiden University, P.O. Box 9555, 2300 RB Leiden, The Netherlands; 2 Leiden Institute for Brain and Cognition, Leiden University, Leiden, The Netherlands; 3 School of Psychology, Cardiff University, Cardiff, UK

**Keywords:** Reflective functioning, Aggression, Sensitivity, Intrusiveness, Infancy

## Abstract

Maternal reflective functioning (RF) has been associated with quality of parent-child interactions and child development. This study investigated whether prenatal RF predicted the development of infant physical aggression and whether maternal sensitivity and/or intrusiveness mediated or moderated this association. The sample consisted of 96 first-time mothers (*M* = 22.57 years, *SD* = 2.13) and their infants (54 % male). Prenatal RF was measured with an interview, maternal behavior was observed during free play at 6 months post-partum, and infant physical aggression was assessed at 6, 12, and 20 months using maternal reports. Multivariate analyses of variance showed that relatively poor prenatal RF was related to relatively high infant physical aggression. These associations were moderated by maternal intrusiveness, with significant differences in physical aggression between RF-groups reportedly only in the absence of intrusiveness. Generally, mothers reported an increase in physical aggression between 6 and 12 months, except when they had both low RF-skills and were relatively less sensitive. It is concluded that prenatal RF is associated with (development of) infant physical aggression, and may be targeted in intervention programs aimed at reducing early physical aggression. Less adequate parenting, however, may counteract the beneficial effects of good RF, or obscure insight into children’s behavioral development.

Early manifestations of physical aggression can be observed from the second half of the first year of life, when infants are first developing the motor ability to direct force against other people (Hay et al. 2011, 2010). At 6 months of age, 15 % of mothers reported clear signs of anger, and a significant number of mothers reported biting or hitting in their infants. While few 6-month-olds react with anger and physical force when a peer takes an object away from them, most 1-year-olds do (Hay et al. 2010). Generally, an increase in aggression can be found in the 2nd year of life (Alink et al. 2006; Nærde et al. 2014; Tremblay et al. 2004). During the first half of the second year, the average use of physical aggression roughly doubles (Hay 2005). In the second part of the year, some report a continued increase (Tremblay et al. 2004), while a temporary decrease has also been reported (Hay 2005).

During toddlerhood a group of ‘early-starter’ children can been identified, who appear to fail or trail behind in developing more sophisticated regulation strategies (e.g., language and other cognitive abilities) to replace physical aggression and who go on showing the most persistent and severe forms of antisocial behavior later in life (Aguilar et al. 2000; Moffitt et al. 2002). However, individual differences in contentiousness (i.e., an increased inclination for expressions of anger and use of physical force during early social interactions) can be observed in 6-month-olds and predict infants’ later use of force towards peers as well as broader conduct problems (Hay et al. 2010, 2014). These findings suggest that some individuals might already set forth on the trajectory to high levels of aggression as early as 6 months of age (Hay et al. 2014).

When high levels of physical aggression are present and/or persist after toddlerhood, negative outcomes including high conflict involvement, poor social skills, academic failure, and internalizing problems are increasingly likely (Campbell et al. 2006). Most longitudinal studies on aggression have focused on the (pre)school age with only few starting in infancy (for a review see, Keren and Tyano 2012). However, given these adverse outcomes, understanding how physical aggression unfolds at its earliest stages is necessary to identify opportunities for prevention and intervention (Côté et al. 2006; Tremblay et al. 2004).

Inadequate parental reflective functioning (RF) and negative parenting behavior (i.e., unresponsiveness, intrusiveness, lack of positive involvement, and hostile or coercive parenting) both have been linked to children’s behavioral problems, whereas adequate parental RF and positive parenting behavior (i.e., sensitivity, involvement, and warmth) have been shown to be a protective factor in this respect (Benbassat and Priel 2012; Edwards and Hans 2015; Ha et al. 2011). Therefore, this study investigated whether maternal prenatal RF can predict the development of infant physical aggression and whether maternal *sensitivity* (i.e., capacity to respond to children’s signals in a contingent, timely and appropriate manner) and *intrusiveness* (i.e., degree to which parents interfere with their child’s needs and behaviors and handle the child forcefully) mediated or moderated this association.

Parental RF or parental mentalizing refers to the parent’s capacity to understand his or her own mental states (defined as beliefs, desires, feelings, intentions, and thoughts), the ability to keep the child’s mental states in mind, and the awareness that an individual’s behavior is a reflection of mental states (Ordway et al. 2015; Slade 2005). During pregnancy maternal RF starts to develop, as the mother-to-be prepares for the birth of the baby by making room for the infant both in mind and in practice (Slade et al. 2009). More specifically, prenatal RF refers to the mother’s ability to think of the fetus, from at least the last trimester onwards, as a separate individual with developing personal features, needs, and temperament (Pajulo et al. 2015). Developmental continuity has been shown regarding pre- and postnatal RF (Arnott and Meins 2008; Steele and Steele 2008).

Parental RF may be especially important during infancy, when the infant’s communication is limited to a non-verbal level, and therefore parents interpret the infant’s internal world through observation of their child’s behavioral and affective cues. The reflective parent’s empathic responses will promote the infant’s sense that emotions are acceptable and manageable, which, in turn, will enable the infant to develop a capacity to self-regulate, and lead to better psychosocial adjustment later in life (Ordway et al. 2015). When parents act on incorrect assumptions about their child’s mental states, this might result in the child feeling confused and being misunderstood by his/her parents. Consequently, the child may become withdrawn, aversive, hostile or coercive (Fearon et al. 2006). Lower postnatal parental RF and maternal mind-mindedness have been associated with more externalizing and internalizing behavior in children (Benbassat and Priel 2012; Ha et al. 2011; Meins et al. 2013), as well as with more attention problems, social withdrawal, and dysfunctional mother-child interaction (Fonagy et al. 2002). Furthermore, more maternal references to mental states have been related to lower scores on reported aggression in 2-year-olds (Garner and Dunsmore 2011), and negative parental perceptions of their child predict ongoing externalizing behavior problems in adolescence (Olson et al. 2000). These findings provide evidence supporting the significant role of maternal postnatal RF in the development of behavioral problems in childhood and adolescence. However, no studies to date have examined the role of prenatal RF in the development of early physical aggression.

Both prenatal and postnatal maternal RF have been related to parenting behaviors (Grienenberger et al. 2005; Pajulo et al. 2008). Better prenatal RF has been linked to more maternal sensitivity and successful positive engagement, and less intrusiveness and internalizing-helplessness behavior 6 months post-partum (Smaling et al. 2016). Higher levels of postnatal RF have been associated with more sensitivity during mother-child interactions (Grienenberger et al. 2005; Pajulo et al. 2008). Furthermore, lower postnatal RF has been associated with more negative maternal parenting behaviors, such as negativity, controlling parenting, and intrusiveness (Stacks et al. 2014). Therefore, maternal RF may be regarded as a critical component for adequate and sensitive caretaking (Fonagy et al. 2002).

Inadequate and unresponsive parenting behaviors (e.g., ignoring child or spanking child with hand when misbehaving) have been related to children’s behavioral problems (Edwards and Hans 2015; Healy et al. 2013; Hughes and Ensor 2006; Keren and Tyano 2012). In contrast, children who have more productive encounters (i.e., more opportunities to engage in activities that are meaningful, challenging, and afford possibilities for learning) are reported to have fewer behavior problems (Benbassat and Priel 2012; Bradley and Corwyn 2005). Similar observations have also been made in very young children (between 1.5 and 3.5 years old), where sensitive parenting was negatively associated with high and increasing levels of physical aggression (Huijbregts et al. 2008), while maternal hostile and ineffective parenting was positively associated with high and rising physical aggression trajectories (Côté et al. 2006; Tremblay et al. 2004).

Reflective parents use their understanding of the child’s mental states to direct their responses towards the child (Slade et al. 2005). These responses are likely to be operationalized through parenting behaviors. Thus, parental behavior could be a mediating mechanism through which prenatal RF exerts its influence on children’s behavioral development. The effects of reflective functioning may also be more prominent when its qualities or characteristics are adequately translated into maternal behavior and interactions with her child (i.e., into sensitivity and a lack of intrusiveness). In other words, it is also possible that moderating effects are present: when a mother has good RF-abilities, but cannot translate these into her behavior (for example, she acts too intrusively), the positive effects of RF may be (partly) decreased.

To our best knowledge, there are no studies that have investigated the potential moderator and/or mediator effects of parenting on the link between prenatal RF and physical aggression. However, there are studies that have examined such effects for the link between postnatal RF and attachment security, which is inversely related to behavioral problems (Grienenberger et al. 2005; Savage 2014). The influence of postnatal RF on infant attachment security was mediated by the maternal ability to regulate infant distress without frightening or otherwise disrupting the child (Grienenberger et al. 2005). Similarly, sensitivity mediated the relation between maternal mind-mindedness (an operationalization of parental mentalizing) and infant attachment (Laranjo et al. 2008), and between postnatal RF and infant attachment (Stacks et al. 2014). Furthermore, postnatal maternal insight - a concept closely related to RF - predicted secure infant attachment, but particularly when mothers also showed sensitivity (Hawkins et al. 2015).

In summary, the first aim of our study was to examine associations between prenatal maternal RF, sensitivity, and intrusiveness, and the development of infant physical aggression at 6, 12, and 20 months. Based on studies so far, we expected better prenatal RF and higher sensitivity to be related to lower infant aggression levels at 6, 12, and 20 months, while higher intrusiveness was expected to be related to higher and increasing levels of infant aggression. Second, we investigated whether maternal sensitivity and intrusiveness mediated and/or moderated the possible link between prenatal RF and infant physical aggression. Based on the previously reported associations between (mostly postnatal) RF, parenting, and physical aggression in young children, it was expected that the effect of prenatal RF on infant physical aggression would in part be explained by maternal sensitivity and intrusiveness. Furthermore, moderating effects were expected with stronger effects of prenatal RF on infant physical aggression when mothers were highly sensitive and low intrusive.

## Method

### Participants

The present study is part of Mother-Infant Neurodevelopment Study in Leiden, The Netherlands (MINDS – Leiden; Smaling et al. 2015). MINDS – Leiden is an ongoing longitudinal study into neurobiological and neurocognitive predictors of early behavioral problems. The study was approved by the ethics committee of the Department of Education and Child Studies at the Faculty of Social and Behavioral Sciences, Leiden University (ECPW-2011/025), and by the Medical Research Ethics Committee at Leiden University Medical Centre Committee (NL39303.058.12), and complied with the Helsinki Declaration and APA ethical standards. Women were recruited during pregnancy via hospitals, midwifery clinics, prenatal classes, pregnancy fairs, and social workers. We oversampled families from a high-risk background in order to obtain sufficient variance in risk factors that might influence children’s early socio-emotional and cognitive development. This was done by collaborating with midwifery/obstetric clinics in areas with a low average social-economic status and/or by recruiting through social workers. Dutch-speaking first-time mothers-to-be between 17 and 25 years old with uncomplicated pregnancies were eligible to participate. We specifically focused on mothers between 17 and 25 years old because they are underrepresented in the current literature. All participating women provided written informed consent.

The total sample at the first assessment (T1), around 27 gestational weeks, consisted of 110 women. 14 families left the study (13 %). Attrition was due to inability to contact (*n* = 7), personal problems (*n* = 4), emigration (*n* = 1), and premature delivery (more than 8 weeks early, *n* = 2). Sample attrition was unrelated to maternal age and ethnicity. However, mothers who dropped out had obtained lower educational levels: *t*(108) = 2.99, *p* < 0.005.

Thus, the final sample for the current study consisted of 96 mother-child dyads who completed all four waves of the study. Women were predominantly Caucasian (84.8 %), 6.2 % Surinamese or Antillean, 4.8 % mixed (Caucasian and other origin), and 4.2 % of other origin. Most women (51.0 %) had completed higher secondary school or lower vocational education, 27.1 % had completed higher vocational education or an university degree, 19.8 % had completed lower secondary school, and 2.1 % completed primary school. Additional maternal demographic variables and infant characteristics are summarized in Table 1.

**Table 1:** Demographic and obstetric sample characteristics

	*M*	*SD*
Maternal age (years)	22.57	2.13
Family monthly income after tax earnings (Euro’s)	2353.15	1190.02
% mothers with a Bachelor’s or Master’s degree	27.1 %	
% Caucasian	84.8 %	
% single mothers	8.3 %	
Infant gestational age at birth (weeks)	39.01	2.00
Infant birth weight (gram)	3344	551
Infant APGAR-score at 5 min	9.43	1.03
Infant sex (% male)	54.2 %	
Mental Development Index (BSID-II)	99.62	18.01
Infant age (months) at T2	5.96	0.41
Infant age (months) at T3	12.15	0.73
Infant age (months) at T4	20.00	0.88
WAIS Vocabulary*	37.32	11.18
WAIS Matrix Reasoning*	19.57	3.62
WAIS Digit Span - backwards*	7.00	2.12

N = 96, M = mean, SD = standard deviation, T1 = first wave, T2 = second wave, T3 = third wave, T4 = fourth wave, *BSID-II* Bayley Scales of Infant Development, 2nd version, *WAIS* Wechsler Adult Intelligence Scale, * = raw scores

### Procedures and Instruments

The assessments at 27 gestational weeks (T1), 6 months (T2), and 20 months post-partum (T4) consisted of a 2- to 2.5-h home visit and were conducted by two female researchers. The third wave (T3) at 12 months consisted of a 1.5- to 2-h lab visit, conducted by one female researcher in the room, while the second researcher was seated behind a one-way screen and made sure all tasks were filmed. T1 included an interview regarding the emotional experience of the pregnancy, a psychiatric interview, and various questionnaires (for more details, see Smaling et al. 2015). After some time to get familiar with the researcher(s), the post-partum waves generally started with several mother-infant tasks with a focus on children’s cognitive and social development. At T2, three subtests of the Wechsler Adult Intelligence Scale, third version (WAIS-III; Wechsler 2005) were administered to the mother. Each wave ended with the mother completing various questionnaires.

#### Reflective Functioning

At T1, the Dutch translation (Smaling and Suurland 2011) of the Pregnancy Interview – Revised (PI-R; Slade 2007a) was administered to assess the level of prenatal reflective functioning. The 22 items of this semi-structured interview tap into the emotional experience of the pregnancy, mother’s prenatal representations of her relationship with her unborn child, and of herself as a parent. The PI-R was digitally recorded and transcribed verbatim. Responses to the individual questions were scored and then, based on the scores per question, the interview as a whole was given a typicality score. This typicality score was used in the analyses. RF was scored on a scale from −1 (negative RF) to +9 (full or exceptional RF), where scores of 5 and above signify distinct evidence of mentalizing (Slade et al. 2007). Markers of high RF are indicated by four different types of reflective capacity: (1) demonstrating an awareness of the nature of mental states, (2) explicitly attempting to tease out mental states underlying behavior, (3) acknowledging developmental aspects of mental states, and (4) mental states in relation to the interviewer (Slade et al. 2007). Transcripts were coded by trained research assistants under supervision of the first author. Prior to scoring, all coders were trained extensively by the first author until intra-class correlations (ICCs) were 0.80 or higher for the individual question scores and overall score using a gold standard set of transcripts. Fifteen percent of the interviews were coded by a second rater. Inter-rater agreement was 0.87 for individual question scores and 0.90 for the typicality score.

#### Maternal Behavior

At T2, maternal parenting behavior was assessed during a 3-min unstructured free play task (FP) in which mothers were given a set of age-appropriate toys and instructed to play with their child as they would normally do. Sensitivity (SEN; degree to which mother appropriately and timely responds to her infant) and intrusiveness (INT; extent to which the mother handles the infant roughly and interferes with the child’s needs and behaviors) were coded using an adapted version of the 4-point global rating scales (0 = absent – 3 = high levels or predominantly present) of the Mother Infant Coding System (Miller et al. 2002). All coders were trained extensively until the ICCs were 0.70 or higher across the dimensions on a subset of 20 recordings. Fifteen percent of the sample was double-coded to assess ongoing inter-rater reliability. ICCs ranged from 0.79 (INT) to 0.84 (SEN). Different coders were used for the coding of maternal behavior and prenatal RF.

#### Infant Aggression

The Cardiff Infant Contentiousness Scale (CICS; Hay et al. 2010) was used at 6 and 12 months to screen for early manifestations of aggression. For this study, the CICS was translated into Dutch and back translated into English, both by the first author and another experienced English-speaking researcher of the MINDS – Leiden team. Mothers were asked to report on infants’ use of physical force in social interactions and the expression of anger, using a 3-point scale ranging from “*not yet*” (0) to “*often*” (2). The CICS has acceptable levels of internal consistency and inter-rater agreement (Hay et al. 2010). Internal consistency was first checked using the initial six items to measure infant physical aggression. To increase internal consistency, the item “pulls hair” was removed resulting in coefficient α = 0.52 at 6 months and α = 0.53 at 12 months, indicating modest measurement precision, an expected finding since the questionnaire consisted of a limited number of items. In contrast to the study by Hay et al. (2010), the removal of item “won’t let go of toys” did not increase the α coefficient, so five items were used.

At 12 and 20 months, the 11-item Physical Aggression Scale for Early Childhood (PASEC; Alink et al. 2006) was used to assess physical aggression. Examples of items are: “bites”, “physically attacks”, and “starts fights”. Mothers were asked whether their child has shown certain behaviors during the past 2 months on a 3-point scale ranging from “*not true*” (0) to “*very true or often true*” (2). At T3, missing values for 4 participants were replaced by the total (standardized) mean score. For the present study, the internal consistency was 0.78 at 12 months and 0.73 at 20 months. This is in line with internal consistency reported by Alink et al. (2006).

#### Infant Cognitive Development

The Infant Mental Development Index (MDI) of the Bayley Scales of Infant Development, 2nd version (BSID-II; Bayley 1993) was used as a global measure of infant cognitive development at T2. The researchers who administered or scored the BSID-II were trained in developmental assessment and interpretation. Raw scores were converted to a scaled score (*M* = 100, *SD* = 15). Internal consistency of the MDI is 0.88 (Nellis and Gridley 1994), with test–retest reliability reported to be *r* = 0.87 and a long term stability of *r* = 0.67 (Bayley 1993).

#### Maternal Cognitive Functioning

Three subtests of the WAIS-III-NL (Wechsler 2005) - Vocabulary, Matrix Reasoning, and Digit Span – backwards - were used as indicators of maternal intellectual functioning at T2. The WAIS-III has repeatedly been reported to provide reliable and valid estimations of intelligence (Wechsler 2005). For each subtest, raw scores were used in statistical analyses.

### Data Analyses

All variables were examined for outliers and violations of specific assumptions applying to the statistical tests used. For each variable, observations with values that exceeded three standard deviations from the mean were recoded to the next highest value within three standard deviations from the mean. Extreme values were recoded for two observations of PASEC at T3 and at T4. First, Pearson correlation analyses were performed to examine associations between infant aggression, prenatal RF, sensitivity, and intrusiveness.

Next, possible mediation effects by maternal sensitivity and intrusiveness of the effects of prenatal RF on infant physical aggression were tested using bootstrap procedures described by Preacher and Hayes (2008).

Subsequently, two groups were created for prenatal RF based on the median, which formed the main between-subjects variable in subsequent analyses of variance. Multivariate analyses of variance (MANOVA) were used to examine possible differences between both RF-groups in infant aggression at 6, 12, and 20 months*.* Two repeated measures analyses of variance (RM-ANOVA) were performed to examine whether reported absolute physical aggression scores changed from 6 to 12 months (using the CICS), and from 12 to 20 months (using the PASEC).

Two RM-ANOVAs with standardized scores at 6, 12, and 20 months (one using CICS at 6 and 12 months, and PASEC at 20 months; and one using CICS at 6 months, and PASEC at 12 and 20 months) were also conducted to examine differences between the RF-groups in the development of infant aggression across three assessment waves. Of note: one can only see whether the two RF-groups developed differently here in standardized units compared to the mean (= 0), so whether the RF-groups developed aggression scores further or closer to the mean of the entire group of children across assessments.

Finally, in order to test potential moderation effects, sensitivity and intrusiveness were dichotomized and added, separately, as independent variables to the MANOVA and RM-ANOVAs predicting infant aggression. All analyses were conducted using the Statistical Package for Social Sciences (SPSS) version 22.0 (IBM Corp., Armonk, NY).

## Results

### Preliminary Analyses

Demographic and obstetric characteristics of the sample are presented in Table 1. Correlations between prenatal RF, sensitivity, intrusiveness, and infant physical aggression are listed in Table 2. Of the three indicators of maternal cognitive ability, only Vocabulary scores were related to prenatal RF (*r* = 0.30, *p* < 0.005), sensitivity (*r* = 0.20, *p* < 0.05), and intrusiveness (*r* = −0.28, *p* < 0.005). However, Vocabulary was not associated with reported infant aggression (neither were maternal scores on Digit Span and Matrix Reasoning). Therefore, these indicators of maternal cognitive ability were not included as potential covariates.

**Table 2:** Correlations between prenatal reflective functioning, infant aggression and maternal behavior

		1	2	3	4	5	6	7
1.	Prenatal RF	1						
2.	T2 aggression (CICS)	−0.17*	1					
3.	T3 aggression (CICS)	−0.05	0.21*	1				
4.	T3 aggression (PASEC)	−0.19*	0.15	0.38**	1			
5.	T4 aggression (PASEC)	−0.19*	0.09	0.31**	0.42**	1		
6.	T2 Sensitivity	0.23*	0.03	0.07	0.08	−0.04	1	
7.	T2 Intrusiveness	−0.15	−0.04	−0.07	0.05	0.07	−0.44**	1
	*Mean*	3.91	3.00^+^	3.96+	2.64^+^	2.83^+^	2.54	0.43
	*SD*	0.90	1.38^+^	1.69+	1.81^+^	2.30^+^	0.64	0.71

** *p* < 0.01; * *p* < 0.05, *N* = 96, ^+^ = unstandardized values, *SD* standard deviation, *RF* reflective functioning, T2 = second wave 6 months post-partum, T3 = third wave 12 months post-partum, T4 = fourth wave 20 months post-partum, *CICS* Cardiff Infant Contentiousness Scale, *PASEC* Physical Aggression Scale for Early Childhood

Maternal age and family income were not associated with infant aggression or maternal sensitivity and intrusiveness. No significant associations were observed between obstetric characteristics and infant BSID-II-scores on the one hand and maternal RF, sensitivity, intrusiveness, and infant aggression on the other. No group differences were found between boys and girls with respect to infant aggression at 6, 12, and 20 months. Furthermore, no differences were found for level of prenatal RF, sensitivity, and intrusiveness between mothers of boys and girls. Therefore, maternal age, family income, obstetric characteristics, infant sex and infant BSID-II-scores were not included as covariates in subsequent analyses.

Prenatal RF was positively associated with sensitivity, indicating that better prenatal RF was associated with more sensitive parenting. For intrusiveness a negative trend was observed (*r* = −0.15, *p* = 0.068). Prenatal RF was negatively associated with infant aggression at 6, 12, and 20 months (see Table 2), indicating that better prenatal RF was related to lower levels of infant physical aggression. Sensitivity was negatively associated with intrusiveness. Sensitivity and intrusiveness were not related to infant physical aggression. Mediation analyses to examine possible indirect effects of prenatal RF on infant aggression through sensitivity and/or intrusiveness could therefore not be carried out.

Furthermore, infant aggression at 6 months was positively associated with infant physical aggression at 12 months (as measured by the CICS, see Table 2). Both measures (CICS and PASEC) of infant aggression at 12 months were moderately correlated. Also, infant aggression at 12 months was positively associated with infant physical aggression at 20 months (as measured with the PASEC).

### Reflective Functioning and Infant Aggression

In order to further examine the effects of prenatal RF on development of infant aggression, two groups were created for prenatal RF based on the median, with mothers with a prenatal RF-score < 4 being assigned to the ‘low-RF’ group (*n* = 35) and those with a RF-score ≥ 4 to the ‘high-RF’ group (*n* = 61).

A MANOVA was used to compare possible differences between both RF-groups in infant aggression at 6, 12, and 20 months. There was a significant multivariate difference between the high and low-RF groups in physical aggression scores: *F*(4,91) = 2.96, *p* < 0.05; Wilk’s Λ = 0.89, partial *η* ^*2*^ = 0.12. Significant univariate differences were observed at 6 months: *F*(1,94) = 4.78, *p* < 0.05, partial *η* ^*2*^ = 0.05, and 20 months: *F*(1,94) = 6.46, *p* < 0.05, partial *η* ^*2*^ = 0.06, indicating that the high-RF group had lower absolute infant aggression scores at 6 and 20 months (*M* = 2.77, *SD* = 1.31 at 6 months, and *M* = 2.39, *SD* = 2.12 at 20 months) compared to the low-RF group (*M* = 3.40, *SD* = 1.44 at 6 months, and *M* = 3.60, *SD* = 2.44 at 20 months). A trend in the same direction was observed for 12 months (*p* = 0.083; low-RF *M* = 3.06, *SD* = 2.20; high-RF *M* = 2.39, *SD* = 1.61, PASEC-scores).

**Fig. 1:**
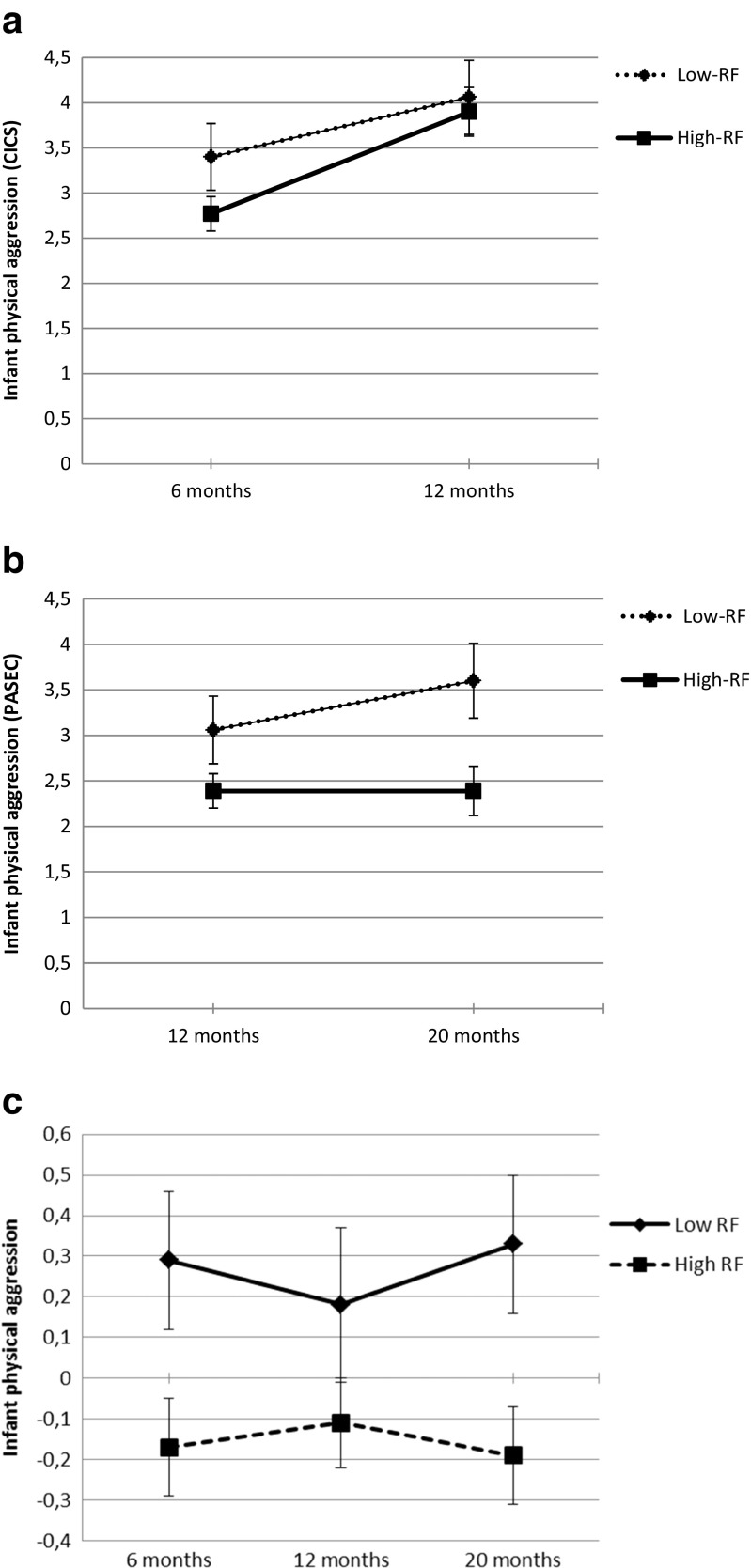
**a**. Infant physical aggression from 6 to 12 months for the group with low reflective functioning (RF) skills versus the group with high RF-skills as measured by the Cardiff Infant Contentiousness Scale (CICS) **b**. Infant physical aggression from 12 to 20 months for the group with low reflective functioning (RF) skills versus the group with high RF-skills as measured by the Physical Aggression Scale for Early Childhood (PASEC). **c**. Standardized infant physical aggression scores across time for the group with low reflective functioning (RF) skills versus the group with high RF-skills using the Cardiff Infant Contentiousness Scale at 6 months and the Physical Aggression Scale for Early Childhood at 12 and 20 months

Next, two RM-ANOVAs were performed to examine whether reported absolute physical aggression scores changed from 6 to 12 months (using the CICS, see Fig. 1a), and from 12 to 20 months (using the PASEC, see Fig. 1b). The first RM-ANOVA, with CICS-aggression scores at 6 and 12 months as dependent variables, revealed a significant effect for Time: *F*(1,94) = 18.77, *p* < 0.001, partial *ŋ* ^*2*^ = 0.17, indicating an increase in reported infant physical aggression between 6 and 12 months. There was no significant Group by Time effect, indicating that reported physical aggression increased for both the low and high RF-groups. The second RM-ANOVA, with PASEC-aggression scores at 12 and 20 months as dependent variables, showed no significant effects of Time or Group by Time. The significant difference between the high and low-RF group: *F*(1,94) = 6.83, *p* < 0.01, partial *ŋ* ^*2*^ = 0.07, indicated higher aggression levels across time for the low RF-group (and therefore largely confirmed results of the MANOVA).

Next, two separate RM-ANOVAs (one with the CICS-score and one with the PASEC-score at 12 months) were conducted using standardized scores to examine differences between the RF-groups in the development of infant physical aggression between 6 and 20 months. For both analyses, results showed no significant Time by Group effects, indicating that there were no differences in developmental pattern for the two RF-groups. The overall group difference in physical aggression between the high and low RF-groups was confirmed with both the CICS: *F*(1,94) = 6.39, *p* < 0.05, partial *η* ^*2*^ = 0.06,and PASEC: *F*(1,94) = 10.32, *p* < 0.005, partial *η* ^*2*^ = 0.10] (see Fig. 1c).

### Reflective functioning, maternal behavior, and infant aggression

During free play, 66 women did not show signs of intrusiveness, 22 were minimally intrusive, 6 were mixed or moderately intrusive, and 2 were predominantly intrusive. Fifty-nine women were predominantly sensitive, 31 were mixed or moderately sensitive, and 6 were low sensitive. As the variance and distribution of scores for sensitivity and intrusiveness were limited, dichotomous variables were created for both maternal behaviors. Sensitivity was recoded as low-SEN (SEN-scores ≤2, *n* = 37) or high-SEN (SEN = 3, *n* = 59), whilst intrusiveness was recoded into no-INT (INT = 0, *n* = 66) or some-INT (INT-scores ≥1, *n* = 30).

**Fig. 2:**
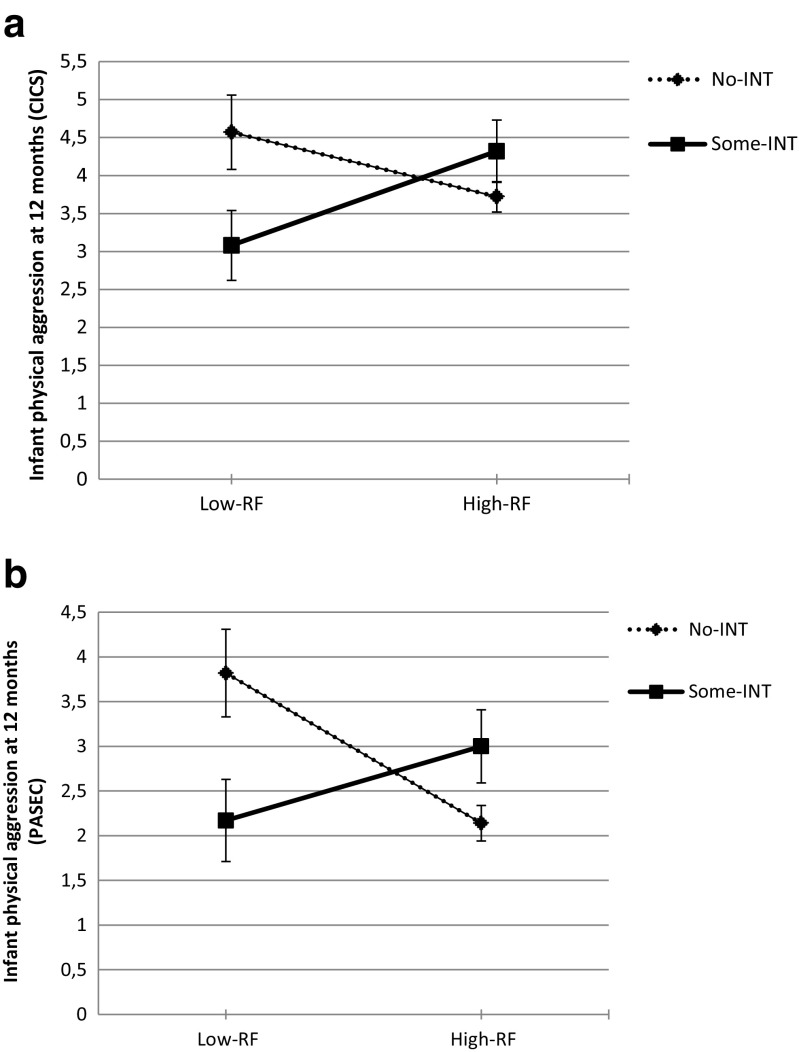
**a**. Moderating effect of intrusiveness (INT) on the association between prenatal reflective functioning (RF) and infant physical aggression at 12 months as measured by the Cardiff Infant Contentiousness Scale (CICS). **b**. Moderating effect of intrusiveness (INT) on the association between prenatal reflective functioning (RF) and infant physical aggression at 12 months as measured by the Physical Aggression Scale for Early Childhood (PASEC)

Two separate MANOVAs were conducted with the dichotomous measures of RF and sensitivity or intrusiveness as independent variables and infant aggression scores as dependent variables. The MANOVA with sensitivity confirmed the effects of RF-group. No effects were observed for SEN or SEN by RF-group. For the MANOVA with intrusiveness, the multivariate tests showed a trend effect for RF-group: *F*(4,89) = 2.03, *p* = 0.097; Wilk’s Λ = 0.92, partial *η* ^*2*^ = 0.08. Also, a RF-group by INT effect was found: *F*(4,89) = 2.92, *p* < 0.05; Wilk’s Λ = 0.88, partial *η* ^*2*^ = 0.12. Between-subjects effects showed a significant RF-group by INT effect for infant aggression at 12 months: *F*(1,92) = 7.82, *p* < 0.005, partial *η* ^*2*^ = 0.08 for the CICS (see Fig. 2a), and *F*(1,92) = 7.94, *p* < 0.005, partial *η* ^*2*^ = 0.08 for the PASEC (see Fig. 2b). A similar trend was observed for 20 months (*p* = 0.088). Additional t-tests to compare infant aggression at 12 months between both RF-groups per INT-group were conducted. For the some-INT group there were no differences between high and low-RF in reported aggression, but in the no-INT group, the low-RF group reported significantly more infant aggression compared to the high-RF group, CICS: *t*(64) = 1.99, *p* < 0.05; and PASEC: *t*(29.80) = 2.60, *p* < 0.05).

The RM-ANOVA to examine whether reported physical aggression changed from 6 to 12 months (using the CICS) with SEN and RF as independent variables confirmed the main effect of Time, indicating an increase in physical aggression between 6 and 12 months: *F*(1,92) = 20.26, *p* < 0.001, partial *ŋ* ^*2*^ = 0.18. Also, a SEN x RF-group x Time interaction was found: *F*(1,92) = 4.03, *p* < 0.05, partial *ŋ* ^*2*^ = 0.04. In the high-SEN group, both RF-groups reported an increase in infant physical aggression from 6 to 12 months, for the low-RF: *t*(18) = 2.44, *p* < 0.05, and for the high-RF: *t*(39) = 3.51, *p* < 0.005. For the low-SEN group, reported physical aggression levels increased in the high-RF group: *t*(20) = 3.20, *p* <0.01, while aggression levels remained stable in the low-RF group (*ps* > 0.6). The RM-ANOVA investigating changes in reported physical aggression from 12 to 20 months (using the PASEC) did not show Time- or SEN-related effects over and above the significant group difference between low and high RF: *F*(1,92) = 5.40, *p* < 0.05, partial *ŋ* ^*2*^ = 0.06.

Next, two similar RM-ANOVA were executed, but with intrusiveness as an independent variable in addition to RF-group. The increase in physical aggression from 6 to 12 months was confirmed: *F*(1,92) = 16.98, *p* < 0.001, partial *ŋ* ^*2*^ = 0.16. The INT x RF-group x Time effect approached significance (*p* = 0.067). The significant interaction between RF-group and INT was confirmed: *F*(1,92) = 5.86, *p* < 0.05, partial *ŋ* ^*2*^ = 0.06. The low-RF group reported significantly more infant aggression compared to the high-RF group, but only when mothers showed no intrusiveness, at 6 months: *t*(64) = 2.33, *p* < 0.05; and at 12 months: *t*(64) = 1.99, *p* < 0.05.

The RM-ANOVA with the PASEC aggression-scores revealed a similar interaction between RF-group and INT: *F*(1,92) = 7.05, *p* < 0.01, partial *ŋ* ^*2*^ = 0.07. Again, the low-RF group reported significantly more infant aggression compared to the high-RF group, but only when mothers showed no intrusiveness, at 12 months: *t*(29.80) = 2.60, *p* < 0.05, and at 20 months: *t*(64) = 3.01, *p* < 0.005. The lack of significant Time x RF-group x INT interactions indicated that the underlying pattern of RF-effects being particularly evident in case if no or little intrusiveness was present across time.

## Discussion

The purpose of this study was to examine the effect of prenatal maternal RF on the development of infant physical aggression, and the possible moderating and/or mediating effects of maternal sensitivity and intrusiveness on this potential association. Prenatal RF was negatively associated with maternal reports of infant physical aggression at 6, 12, and 20 months. Maternal sensitivity and intrusiveness did not mediate the link between prenatal RF and infant physical aggression. Moderating effects for intrusiveness and sensitivity were observed. Better prenatal RF was particularly associated with less infant physical aggression among mothers who showed no or low signs of intrusiveness. However, for mothers who showed some intrusiveness in the interaction with their infant, prenatal RF-level seemed to have little effect on infant physical aggression. Generally, infant physical aggression increased from 6 to 12 months, but mothers with both low prenatal RF and mixed or low sensitivity did not report an increase.

As expected, better maternal prenatal RF was related to lower infant physical aggression levels. Our findings provide evidence supporting the notion that maternal RF plays an important role in children’s social development. It has been suggested that maternal RF stimulates in the child a better understanding of his/her own mind and the minds of others, thereby supporting the developing RF-ability in the child (Sharp and Fonagy 2008). When this ‘meeting of minds’ does not take place in early development, the child seems to be at an increased risk of developing behavioral problems and psychopathology, which may be partly due to impaired mentalizing and less optimal social-cognitive development (Laranjo et al. 2010; Sharp and Fonagy 2008; Slade 2007b). Precursors of mentalizing start to develop late in the first year of life (Onishi and Baillargeon 2005) and should be a future focus of studies investigating RF-behavior associations.

The effect of prenatal RF on reported infant physical aggression was not mediated by maternal parenting behavior. Surprisingly, no significant associations were found between maternal sensitivity and intrusiveness at 6 months and infant aggression at 6, 12, and 20 months. The fact the we (and others, e.g., Leerkes et al. 2009) did not find these links might be related to the time point - 6 months post-partum - used to observe sensitivity and intrusiveness: mixed results exist concerning the degree to which sensitivity and intrusiveness can be regarded as stable factors during infancy and later development (Joosen et al. 2012; Kemppinen et al. 2006; Lohaus et al. 2004). Alternatively, the lack of associations between parenting and infant aggression might be due to the limited distribution in scores for intrusiveness and sensitivity. Mothers in our sample were, on average, relatively highly sensitive and non-intrusive during the free play task. It is possible that more variation in sensitivity and intrusiveness would be observed during tasks other than the relatively short and distress-free task administered here.

A moderating effect of intrusiveness on the link between prenatal RF and the development of infant physical aggression was found. Prenatal RF was negatively associated with infant physical aggression among mothers who showed no or limited signs of intrusiveness. For this group, rudimentary or better prenatal RF was linked to lower levels of infant physical aggression. For mothers who showed some intrusiveness, prenatal RF-level did not predict reported levels of infant physical aggression. These results indicate that beneficial effects of RF are more evident among women who show no intrusiveness. For the more roughly handling and/or interfering mothers other parenting skills or mother and infant factors might play a more important role in the development of infant physical aggression.

Generally, infant physical aggression increased from 6 to 12 months. However, when mothers had both low prenatal RF-skills and showed low or mixed sensitivity during the interaction with their infant, this increase in physical aggression was not reported. As an increase in physical aggression in these 6 months is to be developmentally expected (Hay et al. 2010), the fact that these women did not report such an intensification may indicate that these women are less able to accurately ‘read’ their infant or that they are less focused on their infant, thereby failing to notice certain behaviors. This would also implicate that, especially for this group of women, behavioral observations or more objective instruments are required rather than maternal reports of infant behavior.

Besides sensitivity and intrusiveness, there may be other aspects of parenting through which prenatal RF exerts its influence on children’s aggressive behavior, or that moderate the observed associations. Different, but often related, mechanisms may be involved as well. One example of such a mechanism that has often been studied in the context of reflective functioning is attachment security. Another potentially important mechanism is early social cognition. Early social cognition or precursors to social cognition in the infants/toddlers may be influenced by the social cognitive abilities (e.g., the RF-level) of parents, and this in turn may influence social behavior. All mechanisms and all ‘outcome measures’ (here: physical aggression), may, at least in part, be a reflection of shared genetics. Also, the source and extent of contribution of such genetic propensities in the prediction of physical aggression might change over time in early childhood (Lacourse et al. 2014). The (perinatal) environment created by the mother may also influence physical aggression development through its impact on gene expression and brain development (Ouellet-Morin et al. 2009). Consequently, the relation between maternal prenatal RF and child aggression may also (in part) be accounted for by genetics that are shared by mother and child, although shared genetics might play a more important role in associations between for example mother aggression and child aggression, or maternal RF and child mentalizing abilities. Finally, there is the possibility that interactions between maternal factors and the child genotype exist. A number of genetically-informative studies showed that prenatal risk factors predicted aggressive outcomes in offspring, even when controlling for genetic factors (Rice et al. 2010). For the investigation of (prenatal) RF such designs have not yet been used. This seems an important topic for future research. However, given that there are significant associations between prenatal RF and infant physical aggression, and given the fact that the perinatal environment is (partially) amenable to intervention, the results of this study may be considered important also in the absence of a genetically informative design.

Contrary to our expectations, we only found an increase in infant aggression from 6 to 12 months, but not from 12 to 20 months. This might be due to the restricted age range, as we only looked at early manifestations of physical aggression up till 20 months post-partum. Generally, the first signs of infant aggression can be identified in the first year of life (Nærde et al. 2014; Hay et al. 2011, 2010), whilst the majority of children are exhibiting physical aggression toward siblings, peers, and adults by 17 months (Hay et al. 2000; Keenan and Wakschlag 2000), with a decline in physical aggression from age 3 years onwards (Alink et al. 2006; Tremblay et al. 2004). Thus, further follow-up measures of physical aggression beyond 20 months may be required to observe the expected increase (and subsequent decrease). Regardless of the potential explanations for the lack of an increase in reported physical aggression between 12 and 20 months, our results indicate that at least part of the increase already takes place in the second half-year of life. They also show that even during early infancy individual differences in physical aggression can be predicted based on prenatal maternal RF.

This study has a number of limitations. First, a different measure was used to rate infant aggression at 6 and 12 months (CICS) as compared to 12 and 20 months (PASEC). One limitation of the CICS scale, used at 6 and 12 months, is the fact that only a moderate level of internal consistency was achieved, although this was similar to those obtained in other studies with α-coefficients for aggression items ranging from 0.51 to 0.72 (Côté et al. 2006; Hay et al. 2010; Nærde et al. 2014; NICHD Early Child Care Research Network 2004; Tremblay et al. 2004). Furthermore, the contentiousness items were incorporated into a developmental milestones checklist and presented to the parents as behaviors that all infants might be expected to show. This potentially reduces the effects of social desirability. Following from this, a second potential limitation is that we used maternal report to assess infant physical aggression. Parental perceptions of child functioning may be biased by relationship with the child, knowledge of child behavior, social desirability, emotional status, personality, and inconsistent interpretation of items (Kagan et al. 1994). However, parental perceptions of their child’s aggressive behavior have been differentiated in meaningful and consistent ways (Bates 1990). Also, parents’ ratings of infants’ anger and aggression at 6 months correlate with observed aggression in 12-month-olds (Hay et al. 2011). Nonetheless, use of multiple informants (e.g., partners or co-parents) or direct observation of infant physical aggression would strengthen the findings presented here. Third, mothers who discontinued participation in the study had a slightly lower level of education. This might have resulted in the loss of some more extreme cases. Finally, given that prenatal RF is coded based on verbal narrative, one could imagine that the current RF measurement might not always best capture the true mentalizing capacity of a mother who has difficulty with expressive language.

In order to prevent (continuation of) physical aggression, both risk and protective factors should be targeted, at least as much as the disruptive behaviors themselves (Hughes and Ensor 2006). Maternal RF seems an interesting candidate for incorporation in prevention and intervention programs (Katznelson 2014; Ordway et al. 2014a, b). The primary aim of any RF-based program must be the development of a reflective stance in parents (Slade 2007b). This is based on the presumption that helping mothers develop a reflective stance would enable them to become more sensitive, regulating, and autonomy-promoting parents, resulting in a positive effect on a range of developmental outcomes in the infant (Ordway et al. 2014a, b; Sadler et al. 2013; Slade 2007b). Three areas are of particular interest for interventions aiming to enhance RF during pregnancy: 1) mentalizing about the self as a mother; 2) mentalizing the baby as having a separate mind; and 3) mentalizing about the emerging relationship with the fetus (Markin 2013). There are various strategies to encourage maternal RF (Markin 2013; Sadler et al. 2013, 2006). For example, clinical care and counseling may include the clinician modeling a reflective stance, reviewing recorded mother-infant interactions, and putting emotions that underlie behaviors into words for mother or infant (Slade 2007b). The ability to reflect on the child’s mental states in relation to their behavior may provide parents an outline that can help them learn that, when confronted with child-rearing issues (e.g., tantrums, aggression), trying to understand their child’s mental states can help determine how to respond (Ordway et al. 2014b). The present study underlines this notion by providing evidence for the existence of associations between (prenatal) RF and infant physical aggression over time. Results also showed that RF should not be studied in isolation, as factors such as level of intrusive parenting, but probably other factors as well, have a moderating influence on RF-behavior associations.
